# Retrograde and anterograde signaling in the crosstalk between chloroplast and nucleus

**DOI:** 10.3389/fpls.2022.980237

**Published:** 2022-09-02

**Authors:** Masood Jan, Zhixin Liu, Jean-David Rochaix, Xuwu Sun

**Affiliations:** ^1^State Key Laboratory of Cotton Biology and State Key Laboratory of Crop Stress Adaptation and Improvement, School of Life Sciences, Henan University, Kaifeng, China; ^2^Department of Molecular Biology and Plant Biology, University of Geneva, Geneva, Switzerland

**Keywords:** chloroplast, gene expression, photosynthesis, plastid, retrograde signaling, signal transduction, transcription factor, tetrapyrrole biosynthesis

## Abstract

The chloroplast is a complex cellular organelle that not only performs photosynthesis but also synthesizes amino acids, lipids, and phytohormones. Nuclear and chloroplast genetic activity are closely coordinated through signaling chains from the nucleus to chloroplast, referred to as anterograde signaling, and from chloroplast to the nucleus, named retrograde signaling. The chloroplast can act as an environmental sensor and communicates with other cell compartments during its biogenesis and in response to stress, notably with the nucleus through retrograde signaling to regulate nuclear gene expression in response to developmental cues and stresses that affect photosynthesis and growth. Although several components involved in the generation and transmission of plastid-derived retrograde signals and in the regulation of the responsive nuclear genes have been identified, the plastid retrograde signaling network is still poorly understood. Here, we review the current knowledge on multiple plastid retrograde signaling pathways, and on potential plastid signaling molecules. We also discuss the retrograde signaling–dependent regulation of nuclear gene expression within the frame of a multilayered network of transcription factors.

## Introduction

**FIGURE 1:**
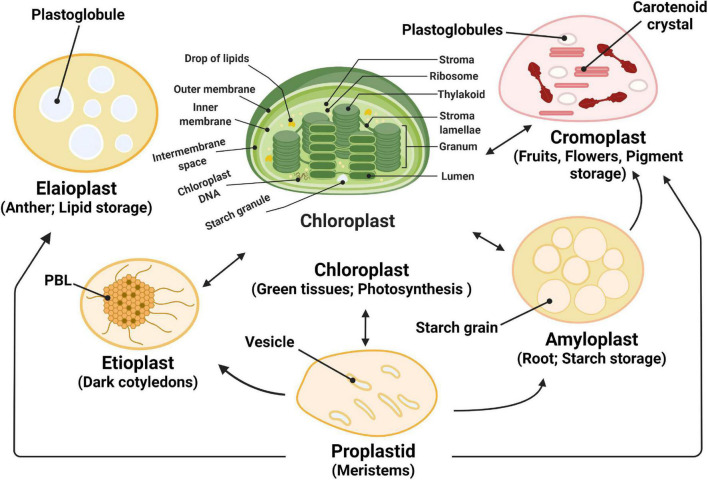
Plastid functions and plastid interconversions. Scheme of the chloroplast with its different compartments. The internal thylakoid membranes fold into stacked grana disks that interconnect via stroma lamellae. Thylakoid membranes enclose the lumen compartment. Plastids exist in different forms, and their identity and abundance are controlled by developmental and environmental cues. Different types interconvert (see the arrows) following a reorganization of the organellar proteome, a process that is controlled by the differentially regulated import of nucleus-encoded proteins. Chloroplasts are photosynthetic plastids, amyloplasts are starch-storing plastids, chromoplasts are carotenoid pigment-accumulating plastids, and proplastids are undifferentiated plastids that can differentiate into the different types of plastids. Etioplasts are chloroplast progenitors that form in darkness and accumulate chlorophyll precursors [in paracrystalline structures called prolamellar bodies (PLB)] that rapidly differentiate upon illumination. Elaioplasts store lipids in lipid droplets known as plastoglobules and exist, for example, in tapetal cells during pollen development.

Photosynthesis, the process through which plants and algae capture light energy and convert it into chemical energy, is one of the most important energy sources to sustain life on earth. In plants and algae, the photosynthetic process occurs in chloroplasts, intracellular organelles with a size of a few microns (Abdallah et al., 2000; Ankele et al., 2007; Yin et al., 2022). Plant viability, seed set, development, and crop yield depend to a large extent on chloroplasts. Photosynthesis has a significant impact on our environment and climate by acting as a major carbon sink and by providing carbohydrates and oxygen for sustaining life (Waters and Langdale, 2009; Cottage et al., 2010). The majority of chloroplasts are localized in plant leaves with only a small portion in plant stems where light absorption is considerably less efficient. Chloroplasts contain their own DNA and protein-synthesizing apparatus. Many key components of the photosynthetic machinery are encoded by the chloroplast genome (Sakamoto et al., 2008; Waters and Langdale, 2009; Yin et al., 2022). Chloroplasts divide by binary fission in a similar way as bacteria (Abdallah et al., 2000; Ankele et al., 2007). They are bounded by the chloroplast envelope consisting of two phospholipid bilayers, the outer and inner membranes. In addition, chloroplasts contain an internal thylakoid membrane system consisting of appressed membrane disks, called grana, connected by stromal lamellae (Sakamoto et al., 2008; Tano and Woodson, 2022). These membranes enclose the inner lumen space and contain the photosynthetic complexes that catalyze the primary light reactions of photosynthesis. The chloroplast stroma includes the space between the chloroplast inner envelope membrane and the thylakoid membrane system, and it contains starch granules, ribosomes, soluble enzymes and nucleoids consisting of chloroplast DNA and associated proteins (Figure 1; Sakamoto et al., 2008).

## Evolution and development of chloroplasts

### Origin of chloroplasts

It is well accepted that chloroplasts originated more than a billion years ago from cyanobacteria which invaded a primitive eukaryotic cell through endosymbiosis (Abdallah et al., 2000; Ali et al., 2022). This event was followed by a massive gene transfer from the endosymbiont to the host genome (Reyes-Prieto et al., 2007; Arsova et al., 2010). The majority of the 1000–8000 genes of the ancient cyanobacterial genome (Barczak-Brzyżek et al., 2022), were either lost or relocated to the host nuclear genome with close to 100 genes remaining in the chloroplast genome (Barkan, 2011). It contains genes that code for tRNAs, rRNAs, and proteins involved in transcription, translation, RNA splicing, proteolysis, photosynthesis, and other metabolic activities (Barnes et al., 2005; Prabha et al., 2016).

The components of the photosynthetic complexes are encoded by both the chloroplast and nuclear genomes. As a result, nucleus-encoded components must be imported into chloroplasts after translation in the cytosol (Barnes and Mayfield, 2003; Baruah et al., 2009a), and assembled with their chloroplast-encoded partners into functional protein complexes through a carefully orchestrated crosstalk between these two genetic systems. The number of proteins in the chloroplast has been estimated between 2,100 and 4,500 based on the presence of predicted chloroplast transit peptides (cTP) (Leister and Kleine, 2008; Breeze and Mullineaux, 2022). In addition, a study combining nine separate datasets found that 1808 proteins were trustworthy chloroplast proteins, with 1720 encoded by the nuclear genome (Chehab et al., 2006; Llamas et al., 2022). A close coordination of chloroplast and nuclear gene expression is essential for chloroplast function, growth and adaptation to stress (Yu et al., 2008; Tano and Woodson, 2022).

### Chloroplast development in land plants

Plants and algae harbor many double-membrane organelles, called plastids, which are derived from undifferentiated non-photosynthetic organelles, called proplastids. Plastids are classified based on their biological functions, in amyloplasts, involved in starch storage, etioplasts, formed in dark-grown leaves that accumulate chlorophyll precursors; chloroplasts, responsible for photosynthesis and biosynthesis of metabolites; chromoplasts that produce carotenoid pigments; elaioplasts with high amounts of plastoglobuli that are used for lipid storage (Figure 1; Chehab et al., 2006; Chen et al., 2008; Sakamoto et al., 2008).

The chloroplast development process is extremely complicated, differing not only between monocotyledonous and dicotyledonous species, but also between developmental stages, organs, and plant tissues, with many underlying mechanisms still unknown (Pogson et al., 2015). Overall, chloroplast biogenesis necessitates the coordination of several biological processes, such as protein and metabolite synthesis and formation of the thylakoid membrane system. The identity and origin of signals that initiate and govern these processes are still largely unknown. Chloroplast development has been shown to have a wide range of effects on plant vitality, seed set, and growth (Abdallah et al., 2000; Pogson et al., 2015). Even when chloroplast formation occurs normally in true leaves, poor chloroplast development at the very earliest cotyledon stages has a detrimental impact on plant growth and yield. Understanding the regulatory mechanisms that govern chloroplast growth is therefore critical (Sakamoto et al., 2008; Cottage et al., 2010; Pogson et al., 2015).

When the leaf primordia develop from the shoot apical meristem (SAM) in true leaves, chloroplasts develop directly from proplastids (Charuvi et al., 2012). The number of proplastids in meristem cells is normally between 10 and 20. These proplastids have a diameter of 0.2–1.0 μm and contain only a few internal membrane vesicles (Sakamoto et al., 2008) formed from either the inner envelope or derived from parental proplastids (Sakamoto et al., 2008). These vesicles differentiate into thylakoids after the perception of light. Chloroplasts are fully developed in mesophyll cells with a significant increase both in their number (20–100) (Lopez-Juez and Pyke, 2005; Schuhmann and Adamska, 2012) and size (5–10 μm) compared to proplastids. This increase in size is likely caused by the newly developed thylakoid membranes and the accumulation of photosynthetic proteins and lipids (Mullet, 2003).

Chloroplasts can also differentiate from etioplasts, an intermediate plastid form in the cotyledons of dark-grown plants. Etioplasts are also present in newly emerged leaves when the plants are placed in the darkness or in the seedlings when they emerge from the earth (Pogson et al., 2015). De-etiolation is the process of differentiation from etioplast to chloroplast upon exposure to light. In contrast to proplastids, which contain vesicles and no differentiated structures, etioplasts feature a prolamellar body (PLB), which has a lattice-like membranous structure. Several essential photosynthetic proteins, such as non-chlorophyll binding photosystem II (PSII) subunits, cytochrome *b*6*f* complex, ATP synthase and ribulose-1,5-bisphosphate carboxylase/oxygenase (Rubisco) have been detected in etioplasts although generally in much lower amounts compared to mature chloroplasts (Escoubas et al., 1995; Dietz et al., 2010; Enami et al., 2011; Estavillo et al., 2011; Dogra et al., 2022). After the disassembly of PLB and the onset of chlorophyll production in response to light exposure, thylakoid membranes develop. During the de-etiolation process, photosystem I (PSI) activity can be detected after only 15 min of light exposure, and PSII and ATP synthase activity after 2–3 h (Finkelstein et al., 1998; Estavillo et al., 2011; Dogra et al., 2022). Biogenesis of chloroplasts is completed in 6–24 h (Fischer et al., 2012; Fisher et al., 2022). In rare circumstances, such as in regreening of Valencia oranges, chloroplasts can differentiate from chromoplasts. This is accompanied by a reduction in the size and quantity of osmiophilic globules, as well as the appearance of well-developed grana (Abdallah et al., 2000; Fisher et al., 2022). Chloroplast development is highly complex and involves many different biological processes. This organelle must be able to sense and act on signals from many different light and metabolic pathways.

## Light signaling pathways control chloroplast development

One of the most important triggers for initiating chloroplast biogenesis is light. In this part, we will cover light signaling mechanisms that underpin chloroplast development.

### Repressors of chloroplast development in darkness

In the dark, germinated seedlings undergo skotomorphogenesis, in which the hypocotyl is extended at the expense of leaf development, an effective strategy for accessing light. The tightly folded apical hook, which helps the seedling to break through the earth crust and protects the unfolded cotyledons as well as the meristem before their emergence from the soil, is another hallmark of this stage. This process ensures that the few nutrients stored in the seeds are used efficiently during the search for light which is essential for photoautotrophic life (Chen et al., 2010; Kami et al., 2010). PHYTOCHROME-INTERACTING FACTORS (PIFs) are a family of basic helix-loop-helix (bHLH) transcription factors that serve as important modulators of the skotomorphogenesis pathway (Leivar and Quail, 2011). Altogether, eight PIFs have been identified, including PIF1, PIF3 to PIF8, and PIF3-LIKE1 (PIL1) which have overlapping and antagonistic roles in skotomorphogenesis (Leivar et al., 2008; Luo et al., 2014). PIF1, PIF3, PIF4, PIF5, and PIF7, for example, stimulate hypocotyl development by activating genes involved in the biosynthetic pathway of the growth hormone auxin (Shin et al., 2009; Stephenson et al., 2009; Li et al., 2012). Moreover, PIF1, PIF3, PIF4, and PIF5 impede chloroplast development by repressing expression of nucleus-encoded photosynthetic proteins (Stephenson et al., 2009; Kim et al., 2011).

Besides preparing for light perception, dark-grown seedlings must deal with soil-induced mechanical stress as the hypocotyl lengthens to emerge from the earth (Liu et al., 2017). ETHYLENE-INSENSITIVE3 (EIN3) which is stabilized by inhibiting the activity of EIN3 BINDING F-BOX 1 and 2 (EBF1/2), is induced by mechanical stress (An et al., 2010; Merchante et al., 2015). EIN3 reacts to mechanical stress by stimulating the expression of a number of downstream genes, including *ETHYLENE RESPONSE FACTOR* (*ERF1*), which is important for reinforcing the hypocotyl cell wall and decreasing the pace of hypocotyl elongation (Maier et al., 2011; Zhong et al., 2014). Furthermore, the EBF1/2-EIN3 pathway activates *HOOKLESS1* (*HLS1*) to regulate seedling apical hook development (Woodson et al., 2013; Llamas et al., 2022). It was found that EIN3 has a similar function as PIF3 and that the two proteins interact to suppress chloroplast growth in the dark. EIN3-PIF3 is able to repress protochlorophyllide (Pchlide) production while also upregulating *PROTOCHLOROPHYLLIDE OXIDOREDUCTASE* (*PORA and PORB*) (Masuda, 2008; Melonek et al., 2010; Maier et al., 2011; Maruta et al., 2012). As a result, EIN3-PIF3 is crucial for regulating Pchlide and *PORA/PORB* levels during de-etiolation to protect the seedling from photooxidative damage. EIN3 and PIF3 have been shown to bind to the promoters of *LIGHT-HARVESTING CHLOROPHYLL A/B-BINDING* (*LHC*) genes, and to repress numerous *LHCA* and *LHCB* genes (Mochizuki et al., 2010; Mittler et al., 2011). Overall, EIN3-PIF3 may be able to control chloroplast development by tying the mechanical and light signaling pathways together. There are a few repressors, including the CONSTITUTIVE PHOTOMORPHOGENIC/DE-ETIOLATED/FUSCA (COP/DET/FUS) proteins, which form three distinct protein complexes and modulate the stability of positive transcription factors throughout the greening process (Mochizuki et al., 2008; Schwechheimer et al., 2022). In *Arabidopsis thaliana* (*Arabidopsis*), *cop/det/fus* mutants have very clear phenotypes. Their seedlings display light-grown phenotypes in darkness, and they accumulate high levels of anthocyanin (Palmer, 1985; Op den Camp et al., 2003; Mullineaux et al., 2006; Nott et al., 2006; Noordally Zeenat et al., 2013). In the dark, COP1, an E3 ubiquitin ligase, uses the ubiquitin-26S proteasome system to degrade several light-responsive transcription factors such as ELONGATED HYPOCOTYL 5 (HY5), HY5 HOMOLOGUE (HYH), LONG HYPOCOTYL IN FAR RED (HFR1), LONG AFTER FAR-RED LIGHT 1 (LAF1), and EBF1/2 (Paul and Foyer, 2001; Pesaresi et al., 2006, 2009, 2011; Patel and Berry, 2008; Barczak-Brzyżek et al., 2022). Other COP/DET/FUS proteins, COP9, DET1 as well as COP10, are also required for the proteasomal degradation of light-responsive regulators in the dark (Pesaresi et al., 2007, 2011).

### Photoreceptors transmit light information to signaling pathways that control chloroplast development

When a seedling emerges from the earth, light triggers a variety of physiological responses, including hypocotyl growth inhibition, chlorophyll accumulation, and chloroplast development. Massive transcriptional reprogramming of both the nuclear and plastid genomes, as well as the onset of a complex signaling interconnecting network, accompany these morphological changes in response to light. This process is called photomorphogenesis and it includes numerous and poorly understood light-sensing mechanisms (Kusuma and Bugbee, 2021).

Plants possess a large number of photoreceptors to sense light intensity, direction, and wavelength. According to their absorption spectrum, these photoreceptors may be divided into different groups: one UV-B light receptor (UVR8) in *Arabidopsis*, three cryptochromes (CRY1-CRY3), two phototropins (PHOT1 and PHOT2), and three Zeitlupe family (ZTL) proteins detecting UV-A/blue light, and five phytochromes (PHYA-PHYE) absorbing red (R)/far-red (FR) light (Kleine et al., 2003; Chory, 2010). These light receptors interact in synergistic, antagonistic, and redundant ways with each other (Mazzella, 2001; Pfannschmidt and Liere, 2005; Pfannschmidt, 2010; Kusuma and Bugbee, 2021).

Light is primarily sensed by phytochromes and cryptochromes during photomorphogenesis. Phytochromes contain the linear tetrapyrrole phytochromobilin as their chromophore. PHYs are found in the cytosol in their inactive Pr form in the dark. They are photoconverted into the active Pfr form when exposed to light. This light-induced conversion is followed by translocation of PHYs from the cytosol to the nucleus, where they are directed to subnuclear sites known as the photobodies (Pfannschmidt et al., 2001; Piippo et al., 2006; Pesaresi et al., 2007; Van Buskirk et al., 2012, 2014). Photobodies are sites for protein degradation and/or transcription factors, as they colocalize with a variety of transcriptional regulators and their E3 ubiquitin ligases as well as photoreceptors (Possingham, 1980; Pogson et al., 2015). Photobodies change in size and quantity in response to changes in light, developmental phases, and daily rhythms (Possingham, 1980; Pesaresi et al., 2007; Van Buskirk et al., 2014; Kusuma and Bugbee, 2021).

Numerous essential light-signaling components, including many transcription factors such as PIFs, EIN3, and COP1, are regulated by PHYs. All PIFs interact physically with the PHYs in their Pfr forms and are subsequently phosphorylated, ubiquitinated, and degraded. PIF phosphorylation has been linked to casein kinase II (CK2), brassinosteroid insensitive 2 (BIN2), photoregulatory protein kinases (PPK1–PPK4), and phytochromes. The full intricacies of light-induced phosphorylation of PIFs, however, have yet to be uncovered (Pfalz et al., 2006; Pham et al., 2018). PIFs are ubiquitinated by distinct E3 ligases after phosphorylation; for example, cullin4 (CUL4) and CUL3-based E3 ubiquitin ligase ubiquitinate PIF1 and PIF3, respectively. The 26S proteasome system then degrades ubiquitinated PIFs (Wostrikoff et al., 2004; Pham et al., 2017). PHYB may facilitate chloroplast growth by promoting connections between EBF1/2 and EIN3, resulting in EIN3 degradation by the 26S proteasome (Schwenkert et al., 2022). Furthermore, by reducing the interaction between COP1 and SUPPRESSOR OF PHYA-105 (SPA) proteins, PHYA and PHYB are able to deactivate COP1 and prevent its proteasome-mediated repression of photomorphogenesis (Pham et al., 2018; Schwenkert et al., 2022).

Cryptochromes are photolyase-like flavoproteins with a flavin adenine dinucleotide (FAD) chromophore (Nott et al., 2006; Pogson et al., 2015; Liang et al., 2022). A CRY photoexcitation model has been proposed in which FAD is postulated to have three redox states: oxidized FAD, semi-reduced FAD or FADH, and completely reduced FADH or FADH2. The photoexcitation cycle of FAD in *Arabidopsis* begins with the ground state, oxidized FAD in darkness, and ends with the semi-reduced FADH upon perception of blue light (Liu et al., 2017). Although this model has been questioned, it is generally agreed that photoexcited CRY adopts an open shape, which is accompanied by metabolic processes such phosphorylation. Various CRY-interacting proteins bind to the altered CRY, establishing a CRY-partner complex, which causes changes in gene expression and developmental programs (Possingham, 1980; Raghavendra and Padmasree, 2003; Pogson et al., 2015). CRY2 also undergoes photodimerization to become active, a process blocked by the BLUE- LIGHT INHIBITOR OF CRYPTOCHROMES 1 (BIC1) protein (Xu et al., 2022). CRY1 and CRY2 control the expression of 5–25% of genes in *Arabidopsis* in response to blue light at the transcriptional or post-translational level (Rodermel and Park, 2003; Ramel et al., 2012; Llamas et al., 2022). CRY2 binds directly to the DNA region shared by PIF4 and PIF5, enhancing low blue light (LBL) induced hypocotyl development at the transcriptional level (Pogson et al., 2015). CRYs may also exert an indirect effect at the post-translational level (Rodermel and Park, 2003; Pedmale et al., 2016; Lee et al., 2022).

Moreover, expression of numerous nuclear genes involved in plastid transcription and photosynthesis, such as *SIGMA FACTOR 5* (*SIG5*), *CHLOROPHYLL A/B-BINDING PROTEIN* (*CAB*), and *RIBULOSE BISPHOSPHATE CARBOXYLASE SMALL SUBUNIT* (*RBCS*), is controlled by CRYs in response to blue light (Rodermel and Park, 2003; Rook et al., 2006; Ruckle et al., 2012). Apart from PIFs, SPA1, and COP1 mentioned above, there may be additional proteins that interact with CRYs to mediate the light signaling pathway. It will be interesting to identify these proteins and to elucidate their function in the CRY signaling pathway that controls chloroplast biogenesis (Schröter et al., 2010; Ruckle et al., 2012; Pedmale et al., 2016; Pham et al., 2018; Liebers et al., 2020).

### Nuclear components involved in the regulation of chloroplast development

When seedlings are exposed to light, the expression of approximately 35% of the nuclear genes encoding chloroplast proteins are changed (Schwechheimer et al., 2022; Schwenkert et al., 2022). Expression of several chloroplast-targeted proteins which are critical for chloroplast development, is considerably increased. These proteins are subsequently transported into chloroplasts where they participate in several chloroplast activities. Anterograde signaling from nucleus to chloroplasts is a process that involves both positive and negative transcriptional regulators from a variety of TF families, including basic leucine zipper (bZIP), Zinc-finger, GARP, and others. In the next part, we will discuss the involvement of these transcription factors in chloroplast biogenesis. A leucine zipper dimerization motif and a DNA binding domain are found in the bZIP transcription factors. According to their DNA-binding region and other conserved motifs, they are divided into 10 groups A-I and S in *Arabidopsis*. Members of the same family have comparable DNA-binding motifs, implying that they bind to similar *cis* regions and, as a result, are functionally redundant, a feature which prevents the appearance of distinctive phenotypes for single mutants (Jakoby et al., 2002; Sherameti et al., 2002; Seo et al., 2008; Pedmale et al., 2016).

HY5 is a well-known factor with a bZIP motif that belongs to the H group. It is able to bind to the promoter region of over 3000 genes and to control a wide range of developmental processes (Jakoby et al., 2002; Lee Keun et al., 2007; Zhang et al., 2011). HY5 was shown to repress the cell elongation genes by directly binding to the G-box (CACGTG) present in their promoter regions (Oyama et al., 1997). HY5 also controls the expression of genes involved in chlorophyll biosynthesis and photosynthesis-associated nuclear genes (*PhANGs*), such as *GUN5, PORC, LHCA4, LHCB1.1*, and *LHCB1.3* (Toledo-Ortiz et al., 2014). The roots of *cop1* and *det1* mutants, in which HY5 inhibition is loosened, become green due to a large accumulation of chlorophyll (Nott et al., 2006; Lee Keun et al., 2007; Stern et al., 2010; Pedmale et al., 2016; Pham et al., 2018).

Furthermore, HY5 promotes thylakoid biogenesis by activating the expression of *DIGALACTOSYLDIACYLGLYCEROL SYNTHASE1 (DGD1)*, and it also plays a role in plastid transcription by activating the expression of *PURPLE ACID PHOSPHATASE 8* (*PAP8*), an essential subunit of the plastid-encoded plastid RNA polymerase (Steiner et al., 2009; Sun et al., 2011). DGD1 is involved in chloroplast lipid metabolism and the DGD proteins can be divided into two types (Lyu et al., 2021). In *Arabidopsis, DGD1* and *DGD2* contain two and one galactosyltransferase domain, respectively (Liu et al., 2004). The function and location of *monogalactosyldiacylglycerol* (MGDG), and DGDG are determined by the structure of the polar head and the fatty acid tails. Moreover, *MGDG* is present in both photosystem I (PSI), photosystem II (PSII), and the cytochrome *b*6*f* complex whereas DGDG is only present in PSII and light-harvesting complex II (Liu et al., 2004; Liang et al., 2019). These two galactosyldiacylglycerols provide biochemical connections between photosynthesis, chloroplast shape maintenance, jasmonate synthesis, phosphate starvation responses, freezing tolerance, and probably other processes (Liu et al., 2004; Lyu et al., 2021).

The G-group members of nuclear bZIP TF family, bZIP16, bZIP68, and GBF1-3, have been linked to various aspects of photomorphogenesis, including photosynthesis, inhibition of hypocotyl elongation, and phytohormone signaling (Surpin and Chory, 1997; Surpin et al., 2002; Sun et al., 2011). *LHCB2.4* has been identified as a target gene of bZIP16, bZIP68, and GBF1 (Surpin et al., 2002; Tano and Woodson, 2022). However, nothing is known regarding the particular role of these genes in the formation of chloroplasts. Functional redundancy and significant physical interactions between these proteins have been discovered previously. Using the yeast two hybrid system, bZIP16, bZIP68, and GBF1, were found to form heterodimers (Tano and Woodson, 2022; Trösch et al., 2022). Such heterodimerizations have also been observed between the GBF1, GBF2, and GBF3 proteins (Pesaresi et al., 2007; Toyokuni et al., 2022; Trösch et al., 2022). GBF1 was also found to interact with other proteins in a variety of ways, including acting antagonistically with HY5 for the expression of *RBCS-1A*, acting additively with HY5 to regulate the expression of *LHCB1.3* (Pesaresi et al., 2007), and acting antagonistically with MYC2, a bHLH TF, in blue light signaling and regulation of hypocotyl elongation (Tyagi, 2001; Tyagi and Gaur, 2003; Pesaresi et al., 2007). Taken together, these findings show that their functional redundancy and diversity as well as their heterodimerization are important for ensuring flexibility to the regulation of this system. Aside from bZIPs, many B-box Zinc Finger proteins (BBXs), including BBX21-BBX25, BBX28, and BBX30-32, play a role in photomorphogenesis. BBX21-BBX23 enhance *HY5* activity, with BBX21 directly binding to the *HY5* promoter and regulating its expression (Tyagi and Gaur, 2003; Jung and Chory, 2010). BBX24, BBX25, BBX28, and BBX32, on the other hand, are negative regulators of HY5 activity, and HY5 might limit the expression of *BBX30* and *BBX31* which also operate as repressors of photomorphogenesis (Holtan et al., 2011; Lin et al., 2018). As a result, BBXs-HY5 act as a critical regulatory network for ensuring optimal photomorphogenesis (Holtan et al., 2011; Gangappa et al., 2013; Lin et al., 2018; Xu, 2020).

**FIGURE 2:**
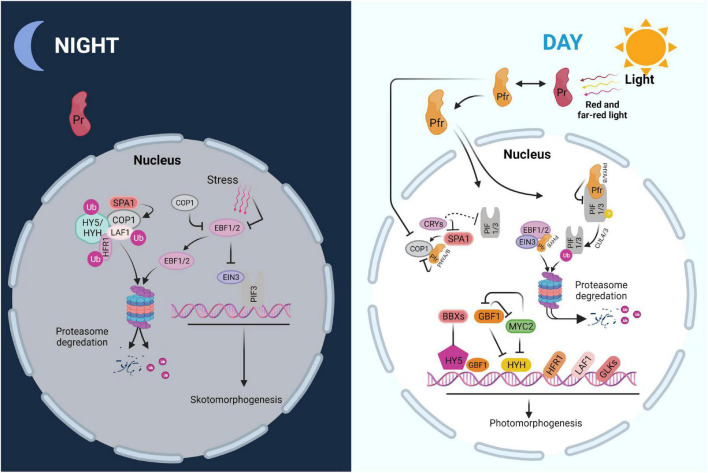
Scheme of major regulatory networks in skoto- and photomorphogenesis. PIF-EIN3 increases skotomorphogenesis and suppresses chloroplast biogenesis in the dark, when combined with soil-induced mechanical stress. COP1, an E3 ubiquitin ligase, degrades EBF1/2, which impedes EIN3 function. Moreover, COP1 degrades numerous light-responsive transcription factors including HY5, HYH, HFR1, and LAF1 via the ubiquitin-26S proteasome system. Photoactivated PHYs in the Pfr form interact with PIFs upon the onset of light, causing their phosphorylation, ubiquitination, and destruction. PHYB may increase EBF1/2-EIN3 interaction, resulting in EIN3 destruction by the 26S proteasome system. PHYs and CRYs deactivate COP1 at the same time, preventing the degradation of positive regulators including HY5, HYH, HFR1, and LAF1. Furthermore, GBF1 and BBX proteins, as well as other regulators such as GLKs, may interact with HY5 to enhance photomorphogenic growth.

Golden 2-like (GLK) proteins, which are part of the GARP superfamily of transcription factors, have also been identified as regulators of chloroplast development. GLKs modulate the expression of *LHCBs*, and genes involved in chlorophyll production directly (Yin et al., 2022). GLK1 and GLK2 control the expression of *DGD1* (see above) (Weissmann and Brutnell, 2012; Yin et al., 2022). In *Arabidopsis*, *glk1glk2* double mutants have a pale green phenotype associated with decreased thylakoid grana (Yasumura et al., 2005; Waters et al., 2009). This phenotype can be compensated by overexpression of either of the two *GLK* genes, suggesting that these two genes have functional redundancy (Waters et al., 2009). Thus, GLK1 and GLK2 appear to play an important role in chloroplast formation. These findings show that the transcriptional response to changes in light quality and quantity and to a shift from darkness to light is governed by a highly coordinated regulatory network (Figure 2).

## Chloroplast transcription systems

The chloroplast DNA (plastome) is around ∼120–160 kb in size and packaged into nucleoids. The nucleoids move from the inner envelope to the thylakoids during chloroplast maturation (Pogson et al., 2015). The plastid genome encodes tRNAs, and rRNAs and key photosynthetic proteins as well as proteins involved in protein synthesis and metabolism. At least two kinds of RNA polymerases transcribe the chloroplast genome. One is nucleus-encoded and consists of a single T3-T7 bacteriophage type subunit (NEP). The other is the plastid-encoded plastid RNA polymerase (PEP), a multi-subunit enzyme of eubacterial type consisting of the core subunits RpoA, RpoB, RpoC1, and RpoC2 that are encoded by plastid genes transcribed by NEP. The PEP complex includes additional bacterial-type nucleus-encoded sigma factors (SIGs) that are imported into the organelle post-translationally. These sigma factors are necessary for targeting the PEP core to the promoter region of defined genes and initiating their transcription. Apart from the sigma factors, PEP is associated to a group of other proteins known as PEP-associated proteins (PAPs), which are likewise nucleus-encoded and required for PEP activity in response to various developmental and environmental signals. In contrast to PEP, NEP can recognize promoters, initiate and terminate transcription on its own as a single catalytic subunit polymerase (Tyagi and Gaur, 2003; Xiao et al., 2012).

Unlike PEP, which can be traced back to cyanobacteria, the evolutionary origin of NEP is unknown. According to current research, the genes for plastid NEP enzymes may have arisen from a duplication of the *RPOTm* gene, which encodes the mitochondrial RNA polymerase (Chan et al., 2014). In monocot plastids, *RPOTp* encodes a single NEP, but in dicot plastids, two NEPs have been described, one encoded by *RPOTp* targeted to plastids and the other by *RPOTmp* targeted to both mitochondria and plastids (Baier and Dietz, 2005; Chan et al., 2014).

In *Arabidopsis*, knocking out one of the NEPs, *RPOTmp* or *RPOTp*, results in reduced pigmentation, altered leaf morphology, and stunted growth whereas the *rpoTmprpoTp* double mutant is seedling lethal (von Gromoff et al., 2006; Xu et al., 2022). NEP and PEP recognize distinct promoter components. The –10 and –35 *cis*-elements, which are comparable to the *E. coli* σ70 promoters, are used by PEP. NEP promoters are divided into three types based on the conserved sequence motifs they recognize: type Ia, type Ib, and type II. The YRTa motif is characteristic for the Ia motif and is conserved in the type Ib motif but with an extra GAA-box. The type II motif, on the other hand, bears little resemblance to the type I motifs (Xiao et al., 2012; Fortunato et al., 2022; Yin et al., 2022).

The involvement of NEP and PEP in the transcription of chloroplast genes has been intensively investigated. Based on their transcription, chloroplast genes may be divided into three categories: genes transcribed exclusively by NEP or PEP, and genes transcribed by both polymerases. NEP transcribes only a few genes, including some housekeeping genes and *rpo* genes required mostly at the beginning of chloroplast biogenesis, while PEP transcribes the vast majority of plastid genes (∼80%) in mature chloroplasts (Fortunato et al., 2022). This is consistent with the finding that in the absence of functioning PEP, accumulation of transcripts from photosynthesis-related genes is dramatically decreased (Jung and Chory, 2010; Breeze and Mullineaux, 2022; Fortunato et al., 2022). However, differential RNA sequencing (dRNA-seq) has revealed that NEP transcribes numerous photosynthetic proteins formerly assumed to be transcribed uniquely by PEP (Jung and Chory, 2010).

Furthermore, examination of run-on transcription activities in PEP-deficient plastids revealed that most plastid genes, regardless of gene class, are transcribed in the absence of PEP (Fortunato et al., 2022), suggesting that NEP alone can transcribe the majority of the chloroplast genome. However, NEP was unable to restore the albino phenotype of PEP-deficient mutants suggesting that the number of RNAs produced by NEP alone is inadequate for normal photosynthetic activity (Jung and Chory, 2010; Fortunato et al., 2022). Chloroplast biogenesis is thus associated with a complex transcription apparatus with some overlap between two RNA polymerases. Both polymerases are required for the establishment of a functional photosynthetic apparatus. Further research is needed to determine the specific role of each polymerase and to elucidate how they interact with other factors for efficient plastid transcription.

### The specific function of the different plastid sigma factors

In *Arabidopsis*, sequence alignment of the six sigma factors (SIG1–SIG6) with their prokaryotic counterparts reveals that all of them belong to the σ70 family of transcription factors (Fischer et al., 2012). SIG2 and SIG6 have been shown to play important roles in chloroplast development, while SIG5 is involved in the blue light-stimulated transcription of *psbD* encoding *PHOTOSYSTEM II REACTION CENTER PROTEIN D* (Heiber et al., 2007; Larkin and Ruckle, 2008; Stern et al., 2010). The other σ factors are less important because corresponding knock-out plants lack visible phenotypes. SIG2 mediates the transcriptional transition from NEP to PEP through transcription of tRNA*^Glu^*, which binds directly to NEP *in vitro* and could limit NEP transcriptional activity (Surpin and Chory, 1997; Stern et al., 2010). The *sig6* mutant, like the *sig2* mutant, is deficient in chlorophyll, especially in the cotyledons (Stern et al., 2010). The levels of many PEP-dependent transcripts, including mRNAs of thylakoid proteins, certain tRNAs, and rRNAs, are decreased in *sig6* seedlings throughout the early stages of development (Pfannschmidt, 2010; Stern et al., 2010; Toyokuni et al., 2022). These findings suggest that SIG6 is especially important during these early phases. The *sig2sig6* double mutant, as predicted, has a more severe pale phenotype and is seedling-lethal, demonstrating critical roles for SIG2 and SIG6 in seedling development (Toyokuni et al., 2022).

### The roles of plastid RNA polymerase-associated proteins during chloroplast biogenesis

Gel filtration and mass spectrometry studies in *Arabidopsis* revealed a total of 35 proteins in transcriptionally active chloroplast DNA-protein complexes (TAC) (Pfalz et al., 2006). Mutants deficient in ten of these TAC proteins exhibit an albino or light green phenotype. T-DNA insertions in the genes of pTAC7 and Mur ligase E domain-containing protein-like (MurE-like) caused a comparable phenotype although they are not physically associated with PEP. They were designated as PAPs based on their phenotype (Pfalz et al., 2006; Grabowski et al., 2008; Fischer et al., 2012). These 12 TAC proteins are known as PAPs because they are thought to be necessary for active PEP transcriptional activity. PAPs were split into three categories based on our present understanding and predictions of their structural domain (Pfannschmidt et al., 2001; Pfannschmidt and Liere, 2005; Piippo et al., 2006; Pfannschmidt, 2010). PAP1/pTAC3, PAP2/pTAC2, PAP3/pTAC10, PAP5/pTAC12, PAP7/pTAC14, PAP8/pTAC6, and PAP12/pTAC7 belong to the first group. This family of proteins binds DNA, RNA, or both, and is thought to play a role in promoter recognition, transcription initiation, and elongation (Nott et al., 2006; Estavillo et al., 2011; Mittler et al., 2011).

Proteins of the second group, PAP4/Fe SUPEROXIDE DISMUTASE 3 (FSD3), PAP6/FRUCTOKINASE-LIKE PROTEIN 1 (FLN1), PAP9/FSD2, and PAP10/THIOREDOXINz (TRXz) are responsible for redox-dependent gene regulation and PEP protection against reactive oxygen species (ROS) (Estavillo et al., 2011; Li and Kim, 2022).

**FIGURE 3:**
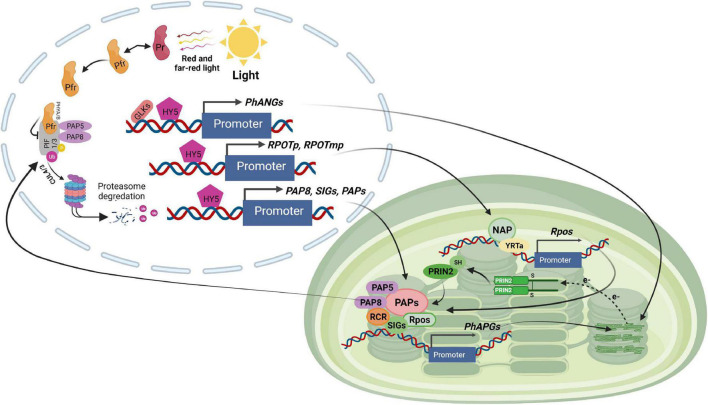
Light signaling pathway controlling plastid transcription. GLKs, HY5 and unknown regulators stimulate the expression of chloroplast proteins encoded by nuclear genes such as *PhANGs, RPOTp*, and *RPOTmp* (encoding NEP), *SIGs* and *PAPs* (encoding PEP components) in response to light exposure. NEP transcribes the *rpo* genes of the PEP core proteins. The PEP core is associated with PAPs, which include PAP5 and PAP8, two dual-localized components implicated in PHY-mediated PIF1 and PIF3 degradation in the nucleus. PEP activity is regulated by other proteins such as PRIN2 in a redox-dependent way. The PEP complex transcribes the photosynthesis-associated plastid-encoded genes (*PhAPGs*). Their products aid in the establishment of photosynthesis when combined with the proteins encoded by *PhANGs.*

The third category includes the remaining PAPs. The function of PAP11/MurE-like is unknown. PAP3 may interact through its carboxyl-terminal region downstream of the S1 domain with several other PAPs including PAP4, PAP7, PAP9, PAP10, and PAP12. PAP3, on the other hand, does not interact directly with any of the PEP core components, such as RpoA and RpoB (Chen et al., 2008; Estavillo et al., 2011). PAP5 and PAP8, two other members of this family, have a dual location in the nucleus and chloroplast where they have distinct roles. PAP5 functions in gene transcription in chloroplasts (Pfalz et al., 2006) and mediates degradation of PIF1 and PIF3 through phytochrome in the nucleus (Galvão et al., 2012; Qiu et al., 2015). PAP5 interacts directly with PAP7 and PAP12 (Yu et al., 2013) although its biological role is unknown. PAP8, like PAP5, plays a crucial role in the creation of photobodies. PAP8 is implicated in the degradation of PIF1 and PIF3 by phytochromes, as well as the stability of HY5 and the GLKs (Figure 3; Liebers et al., 2020). Because of these multiple roles, PAP5 and PAP8 have been proposed to be crucial coordinators of nuclear and chloroplast gene expression during chloroplast biogenesis (Stern et al., 2010; Galvão et al., 2012; Liebers et al., 2020).

Chloroplast gene expression is a complex and highly regulated process, and the molecular relationship between photosynthetic electron transport activity and regulation of plastid gene expression (PGE) has been studied for many years (Rochaix, 2013). A major target of photosynthetic redox signals is the PEP complex. It fine-tunes plastid transcription by sensing redox signals resulting from unbalanced stimulation of both photosystems, in addition to its direct function in plastid transcription (Galvão et al., 2012; Liebers et al., 2020; Fortunato et al., 2022). However the molecular mechanisms underlying this redox control are still unclear. A previous study demonstrated that the redox modulation of PRIN2 activity is responsible for the thiol-mediated regulation of PEP-dependent transcription (Steiner et al., 2011). The photosynthetic electron transport chain is assembled during the greening phase, and after its components are reduced by light, the FTR/TRX system is activated. Through the reduction of the Cys68–Cys68 disulfide link, TRX transforms the PRIN2 dimer to the active monomeric form (Díaz et al., 2018). The monomeric form increases the light-activated transcription of photosynthetic chloroplast genes. As a result, activation of the PEP complex creates a positive retrograde signal that controls expression of the nuclear-encoded photosynthesis genes, therefore synchronizing the activities of the two genomes in response to light (Díaz et al., 2018). The formation of functioning chloroplasts and the initiation of photosynthesis is a complex process that involves several cellular compartments. First, the growth of chloroplasts is light-dependent, and the initial light signal activates photoreceptors, triggering substantial changes in nuclear transcription (Chen et al., 2004; Waters et al., 2009). Second, the expression of the plastid-encoded photosynthesis genes is dependent on the expression and assembly of the nuclear-encoded components necessary for PEP action (Kindgren and Strand, 2015). Using gel filtration and mass spectrometry, the transcriptionally active complex in chloroplasts was shown to comprise 43 nuclear encoded proteins (Kindgren and Strand, 2015). Consequently, given its complex structure, activation of PEP is most likely a rate-limiting step for the onset of photosynthetic gene expression in the plastids. It was proposed that it represents a developmental bottleneck in the establishment of photosynthesis (Pfalz et al., 2015). In addition, the activation of PEP involves a redox regulatory system that modulates PGE (Pfannschmidt and Liere, 2005).

The nature of the retrograde signal is unknown, but it has been suggested that blocked chloroplast development would either disrupt a positive signal emitted by the plastid, which acts in a GUN1-regulated manner, or induce a negative plastid-emitted signal, which represses nuclear transcription in a GUN1-mediated manner (Vinti et al., 2000; Martín et al., 2016). Recent data describing the establishment of photosynthesis during chloroplast development suggest that full *PhANG* induction is dependent on a positive signal from healthy developing plastids (Dubreuil et al., 2018). In addition, it is known that *LHCB* expression is directly correlated with the recovery of PEP activity in the different *prin2-2* complemented lines, suggesting that a positive signal generated by PEP activity in the plastids stimulates LHCB expression in the nucleus. Taken together, the results suggest that the monomeric form of PRIN2 is the active form of the protein, and contributes to the activation of the PEP complex during the establishment of functional chloroplasts in response to light (Díaz et al., 2018). Once PEP is activated, a positive retrograde signal is triggered and thus, the status of the PEP complex links the functional state of the chloroplast to the nucleus, enabling the plant to coordinate expression of photosynthetic genes of the nuclear and chloroplast genomes during seedling development (Díaz et al., 2018).

PAP10/TRX z features a thioredoxin domain and has been shown to interact with PAP6, FLN2, and PLASTID REDOX INSENSITIVE (PRIN2) to mediate the redox control of PEP activity (Figure 3; Waters et al., 2009; Wimmelbacher and Börnke, 2014). It is therefore possible that PAP10 is involved in redox-controlled plastid transcription. PAP4 and PAP9, two members of the second group, both include iron superoxide dismutase domains that protect against oxidative stress during the onset of photosynthetic activity in early development (Myouga et al., 2008; Qing-Bo, 2013). Although the function of the remaining PAPs is unknown, loss of function mutants of all PAPs have a PEP-deficient phenotype characterized by the inability to grow autotrophically and an altered plastid transcription profile (Pfalz et al., 2006; Pfannschmidt, 2010), implying that all PAPs are required for PEP-mediated gene expression.

A surprising finding was that redox-inactive versions of TRXz and FLN1 are able to entirely restore PEP function in the corresponding loss of function mutants during early chloroplast development (Weissmann and Brutnell, 2012; Wimmelbacher and Börnke, 2014; Yin et al., 2022). A possible explanation is that some, if not all, of the PAPs have a structural rather than a regulatory role (Steiner et al., 2011; Liebers et al., 2020; Toyokuni et al., 2022). This suggests that the absence of some PAPs has a negative impact on the structural integrity of the PEP complex during chloroplast maturation. PEP evolved from its cyanobacterial ancestor to associate with numerous PAPs in order to optimize its function in the photosynthetically active chloroplast. The precise functions of the various subunits are currently unknown. The PEP complex is thought to undergo a structural rearrangement during chloroplast development, in which PEP only consists of the core subunits in darkness and is assembled with PAPs upon light exposure (Pfannschmidt et al., 2001; Pfannschmidt and Liere, 2005; Pfannschmidt, 2010). The role of each component and the assembly process will need to be investigated further.

### The roles of (p)ppGpp in chloroplast stress responses

The hyperphosphorylated nucleotides guanosine pentaphosphate and tetraphosphate (together referred to as ppGpp) have emerged as likely candidates for regulating chloroplast stress signaling. They are synthesized from ATP and GDP/GTP by enzymes of the RelA SpoT Homolog (RSH) family in chloroplasts (Boniecka et al., 2017; Field, 2018). In plants ppGpp was shown to be required for the regulation of plant growth and development (Sugliani et al., 2016; Ono et al., 2021), plant immunity (Abdelkefi et al., 2018), and photosynthesis (Sugliani et al., 2016). Many years ago ppGpp was shown to be involved in the stringent response in bacteria which is triggered by nitrogen limitation and results in growth arrest (Cashel and Gallant, 1969). In plants subjected to nitrogen limitation the chloroplast with its photosynthetic machinery represents a major potential nitrogen source for metabolic remobilization. Under these conditions ppGpp was indeed induced and shown to be important for remodeling the photosynthetic electron transport chain, thereby down-regulating photosynthetic activity and providing protection against oxidative stress and cell damage (Romand et al., 2022). Moreover ppGpp was shown to promote the coordinated down-regulation of both chloroplast and nuclear genes of chloroplast proteins and thus to couple chloroplast and nuclear gene expression during nitrogen starvation (Romand et al., 2022). Thus ppGpp appears to be a key regulator of chloroplast activity with a photoprotective role for the adaptation of plants to environmental stress conditions.

## Chloroplast intracellular signaling

In plants, both the plastid and nuclear genomes encode proteins that are essential for photosynthetic function. Most of the chloroplast proteins (∼90%) are encoded by the nucleus (Surpin and Chory, 1997; Surpin et al., 2002; Rodermel and Park, 2003; Barkan, 2011; Ramel et al., 2012; Wu et al., 2019). Many nucleus-encoded gene products interact with plastid-encoded proteins within the chloroplasts to assemble into functional complexes (such as the ATP synthase, PSI, and PSII); others perform regulatory functions within the chloroplasts (such as PPR proteins) (Barkan, 2011). Rubisco, the major CO_2_ fixation enzyme, represents a classic example of gene coordination between nucleus and chloroplast (Wimmelbacher and Börnke, 2014). Each plastid chromosome contains one *rbcL* gene, but each plastome can include up to 100 chloroplast DNA copies, and each photosynthetic plant cell contains up to 100 chloroplasts (Possingham, 1980; Palmer, 1985). The Small subunit (SSU), on the other hand, is encoded by a small group of 5–15 *rbcS* genes in the nucleus (Patel and Berry, 2008). As a result, the number of *rbcL* genes greatly exceeds the number of *rbcS* genes within each plant cell. Despite this large difference in gene copy number, the plastid-encoded *rbcL* gene is coordinated with the nucleus-encoded *rbcS* genes to produce stoichiometric amounts of Large subunit (LSU) and SSU proteins in the chloroplasts, allowing the L8S8 holoenzyme to be assembled. Multiple steps of post-transcriptional regulation are required for the coordinated synthesis of LSU and SSU synthesis, including mRNA stability, RNA processing, translational initiation, translational elongation, protein stability, and final assembly of the L8S8 holoenzyme (Patel and Berry, 2008; Woodson et al., 2013).

Folding of newly synthesized LSU is mediated by the Cpn60/Cpn20 chaperonin complex. This is followed by formation of LSU oligomers consisting of four dimers and by the assembly of the holoenzyme L_8_S_8_, a process which is assisted by the chaperones RbcX, Raf1, and Raf2 (Aigner et al., 2017; Wietrzynski et al., 2021). In both land plants and Chlamydomonas, the stoichiometric expression of the two Rubisco subunits is regulated by the CES process (control by epistasy of synthesis) that represses the translation of LSU in the absence of SSU (Wostrikoff and Stern, 2007). The CES process is also operational during the assembly of the other photosynthetic complexes PSII, PSI, Cytb6f, and ATP synthase in the thylakoid membranes (Choquet and Vallon, 2000), and involves the assembly dependent synthesis of some key subunits of these complexes; it has been mostly studied in Chlamydomonas.

When amaranth seedlings are exposed to different light regimes, translational coordination occurs (Patel and Berry, 2008). In dark-grown seedlings transferred to light (light-shift), the LSU and SSU polypeptides are induced coordinately extremely rapidly with no corresponding increase in their transcript levels. Both *rbcL* and *rbcS* mRNAs are not associated with polysomes in dark-grown plants (when there is no synthesis), but bind to polysomes when these seedlings are exposed to light, allowing both Rubisco proteins to be synthesized coordinately. Moreover, when light-grown plants are shifted to darkness (dark-shift), the synthesis of both Rubisco subunits is promptly arrested. Although Rubisco production is drastically decreased in dark-shifted amaranth seedlings, *rbcL* and *rbcS* transcripts remain bound to polysomes indicating that translation elongation is also regulated in a coordinated manner. These results show that light-induced synthesis of both Rubisco subunits is well synchronized between the two cellular compartments, owing to control at the translational level (Jung and Chory, 2009; Stern et al., 2010).

**FIGURE 4:**
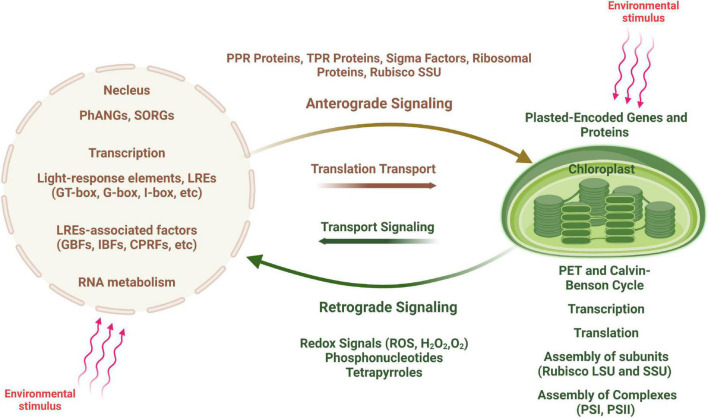
Scheme of anterograde and retrograde signaling in plant cells. An environmental stimulus is perceived by the nucleus, communicated to the chloroplast (shown by red arrows) and leads to changes in chloroplast transcription (anterograde signaling). In contrast, signals generated by the chloroplast are transduced to the nucleus causing changes in nuclear gene expression (retrograde signaling), in particular to regulate the expression of nucleus-encoded photosynthesis associated nuclear genes (*PhANGs*).

There is thus an interactive communication between the two cellular compartments, allowing for the coordinated expression of plastid- and nucleus-encoded photosynthetic proteins (Figure 4). A large number of nuclear encoded factors are involved in this coordination and act at several steps of chloroplast gene expression including transcription, RNA editing, RNA processing and degradation, splicing and translation (Barkan and Goldschmidt-Clermont, 2000). Although nucleus-encoded factors govern most aspects of chloroplast growth and function, communication between the two compartments occurs in both direction, from nucleus to plastids (anterograde signaling) and from plastids to nucleus (retrograde signaling) (Surpin and Chory, 1997; Surpin et al., 2002; Jung and Chory, 2009).

Developmental processes such as leaf maturation and senescence, light irradiance, photosynthetic energy production, redox state, and other factors all affect these signaling networks. Intracellular photosynthetic signaling processes between chloroplasts and nucleus are highly complex, involving the functional integration of components from both inside and outside of these two compartments. Signals from the mitochondria (such as energy and redox state) and the cytosol influence the photosynthetic crosstalk within plant cells (such as transport) (Paul and Foyer, 2001; Barnes and Mayfield, 2003; Strand et al., 2003; Barnes et al., 2005; Stern et al., 2010; Maier et al., 2011; Steiner et al., 2011; Maruta et al., 2012). Protein complexes that control chloroplast transcription, RNA processing and splicing, translation, and assembly/stabilization, as well as pigment-binding complexes participate in these intracellular interactions (Weissmann and Brutnell, 2012; Breeze and Mullineaux, 2022).

### Multiple plastid retrograde signaling pathways

In the plastids of higher vascular plants, four distinct plastid retrograde signaling pathways have been traditionally recognized based on the sources of the signals: Accumulation of tetrapyrrole intermediates, Inhibition of PGE, Changes in plastid redox status, and Production of ROS (Nott et al., 2006; Kleine et al., 2009; Pesaresi et al., 2009; Woodson et al., 2013; Pogson et al., 2015; Garmash, 2022). Several additional plastid retrograde signals have been proposed that include SAL1-PAP (3′-phosphoadenosine 5′- phosphate) and methylerythritol cyclodiphosphate (MEcPP) (Estavillo et al., 2011; Xiao et al., 2012; Liebers et al., 2020).

#### Accumulation of tetrapyrrole intermediates and connection with retrograde signaling

**FIGURE 5:**
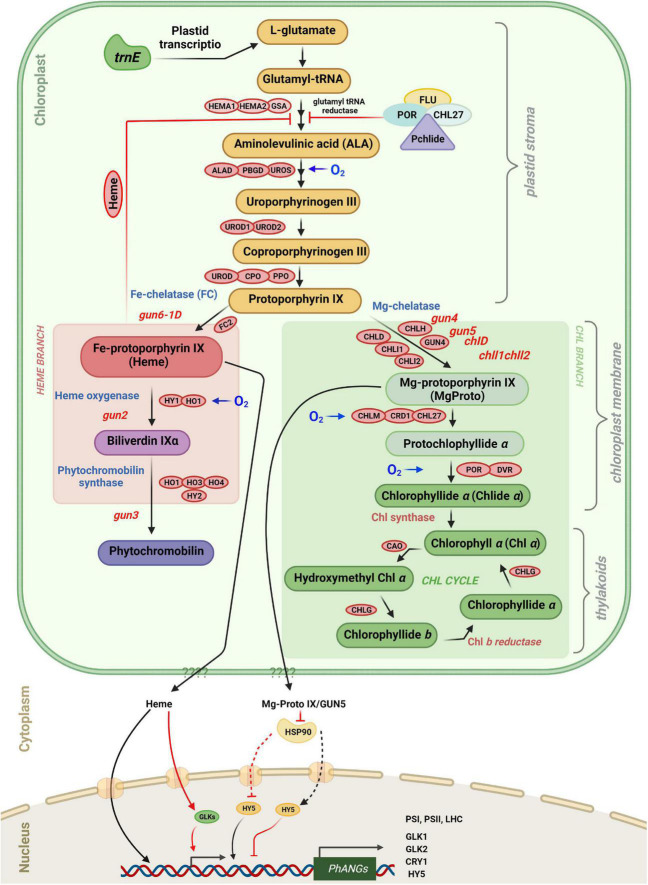
Tetrapyrrole biosynthesis and plastid gene expression with key enzymes. Retrograde signaling is mediated in part by tetrapyrrole intermediates. The different steps in tetrapyrrole biosynthesis are indicated by arrows. FLU as well as the amount of “free” heme control the rate of ALA Synthesis. Protoporphyrin IX can be converted either to Fe-protoporphyrin IX (heme), leading to phytochromobilin, or to Mg-protoporphyrin IX leading to chlorophyll *a* and chlorophyll *b*. The heme branch is mediated by GUN6 (Fe-chelatase), GUN2 (heme oxygenase), and GUN3 (phytochromobilin synthase). Heme acts as a positive regulator of photosynthesis-associated nuclear genes (*PhANGs*), but the nuclear components involved in heme signaling, as well as the precise process by which heme enters the nucleus, remain unclear. GUN5/CHLH and GUN4 (which binds the substrate and product of the Mg-chelatase-catalyzed process and activates the Mg-chelatase) are engaged in the chlorophyll branch. It is proposed that Mg-proto IX is exported from plastids via an unknown route and subsequently binds to HSP90, which in turn represses *HY5* and prevents activation of *PhANG* expression. Enzymes involved in the tetrapyrrole biosynthetic pathway are indicated in red boxes. They are ALA, 5-aminolevulinic acid; HEMA, glutamyl-tRNA reductase; GSA, glutamate-1-semialdehyde 2,1-aminomutase; ALAD, 5-aminolevulinate dehydratase; PBGD, porphobilinogen deaminase; UROS, uroporphyrinogen III synthase; UROD, uroporphyrinogen III decarboxylase; CPO, coproporphyrinogen III oxidase; PPO, protoporphyrinogen IX oxidase; CHLH, Mg-chelatase H subunit; CHLI, Mg-chelatase I subunit; CHLD, Mg-chelatase D subunit; GUN4, regulator of Mg-chelatase; CHLM, Mg-proto IX methyltransferase; CRD1, Mg-proto IX monomethylester cyclase; POR, NADPH:protochlorophyllide oxidoreductase A, B and C; DVR, divinyl-protochlorophyllide reductase; CHLG, chlorophyll synthase; CAO, chlorophyllide a oxygenase; FC, ferrochelatase; HO/HY1, heme oxygenase; and phytochromobilin synthase (HY2).

**FIGURE 6:**
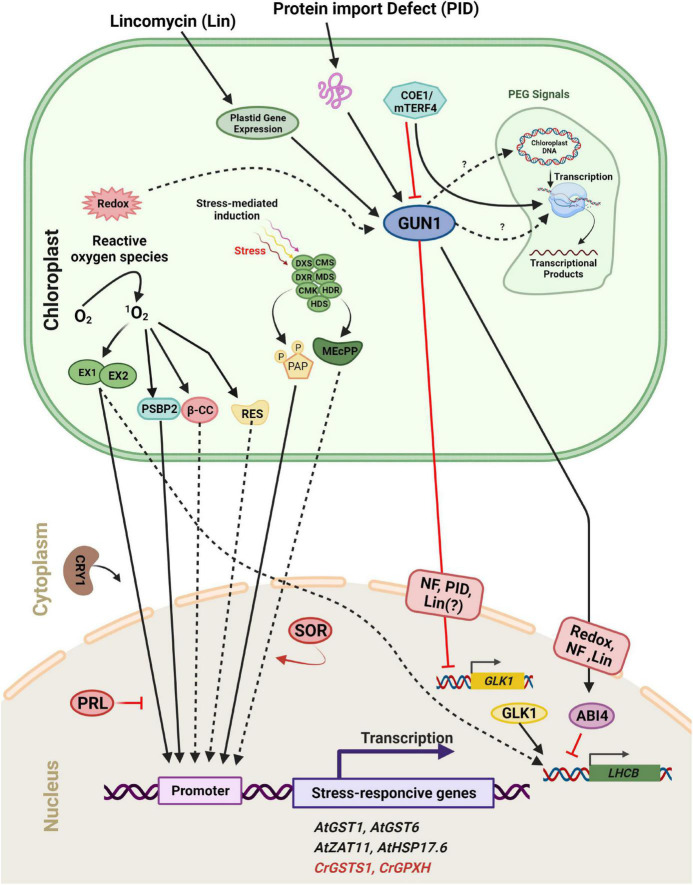
Scheme of plastid retrograde signaling (PGE) in a plant/algal cell. Components are considered to be involved in ^1^O_2_-mediated induction of either *Arabidopsis thaliana* (*At*, black) or *Chlamydomonas reinhardtii* (*Cr*, Red) genes. ^1^O_2_ accumulates during exposure to excess light and activates distinct signaling pathways. The ^1^O_2_ accumulated in the plastid is sensed and/or transmitted to the nucleus via the concerted action of two chloroplast proteins, EXECUTER 1 and EXECUTER 2 (EX1 and EX2). In the cytosol/nucleus, the blue-light photoreceptor CRY1 is involved in ^1^O_2_-mediated stress responses, but PRL may act as a general negative regulator of ^1^O_2_ signaling. One product of the ^1^O_2_ -caused oxidation of carotenoids, β-cyclocitral (β-CC), may act as a second messenger involved in the ^1^O_2_ signaling pathway in plants. A second stress response involves the induction of methylerythritolcyclo diphosphate (MEcPP) and phosphoadenosine phosphate (PAP), two important metabolites that participate in retrograde signaling through chromatin remodeling and changes in nuclear gene expression. Full arrows indicate activation or conversion, and dashed arrows indicate diffusion or transport of molecules. Furthermore, distinct plastid processes [redox change, plastid gene repression by Lincomycin (Lin), Norflurazon (NF), and Protein Import Defect (PID)] generate plastid signals. These signals converge at GUN1 within the plastid. Subsequently, the signal is sent to two transcription factors, ABI4 and GLK1. GUN1 activates ABI4, a negative regulator of *LHCB* expression and also down-regulates the expression of *GLK1*, leading to a subsequent decrease in *LHCB* expression.

Retrograde signaling from the plastid to the nucleus is important for two reasons. First, functional multiprotein complexes in plastids, such as photosystems, are composed of subunits encoded by both the nuclear and plastid genomes whose expression needs to be coordinated. Second, metabolic activities and the functional state of plastids are affected by the external environment, such as light conditions. To adapt plastids must sense the environmental changes and adjust expression of nuclear genes of chloroplast proteins and the corresponding protein flow (Jung and Chory, 2010; Kindgren et al., 2011). Here we focus on the tetrapyrrole pathway and the mechanisms by which changes in the functional or metabolic state of the plastids elicit signals that affect nuclear gene expression. Analysis of perturbations of the tetrapyrrole biosynthetic pathway in the *gun* mutants have provided clues for potential plastid signals (Figures 5, 6) (Susek et al., 1993; Rodermel and Park, 2003; Xiao et al., 2012). Chlorophyll, heme, siroheme, and phytochromobilin are generated by the tetrapyrrole biosynthetic pathway in plastids (Tanaka and Tanaka, 2007; Mochizuki et al., 2008). In land plants the chlorophyll biosynthetic pathway begins with glutamyl tRNA and can be separated into three parts: from ALA to protoporphyrin IX in the chloroplast matrix; from magnesium protoporphyrin to chlorophyllide on the chloroplast envelope membrane; and ultimately Chl *a* and Chl *b* are synthesized on the thylakoid membrane (Figure 5; Matringe et al., 1992).

The first committed step of tetrapyrrole synthesis is the conversion of glutamyl-tRNA to 5-aminolevulinic acid (ALA) by glutamyl-tRNA reductase (GluTR) and glutamate 1-semialdehyde aminotransferase (GSAT) (Figure 5; Tanaka and Tanaka, 2007). In the dark Pchlide cannot be converted to chlorophyllide because the NADPH:Pchlide oxidoreductase (POR) requires light to convert Pchlide *a* to chlorophyllide *a*. Pchlide forms a complex with GluTR and CHL27 (a component of Mg-protoporphyrin monomethylester cyclase3) and the FLUORESCENT (FLU) protein which inhibits GluTR thereby preventing accumulation of free Pchlide which is phototoxic (Kauss et al., 2012; Hou et al., 2019; Abbas et al., 2022). The steady-state level of Pchlide in the *flu* mutant is unrestrained under moderate hypoxia compared to normoxia and hyperoxia, demonstrating that accumulation of Pchlide in various species is also regulated through oxygen sensing. Furthermore, GluTR acts at the rate-limiting step in the tetrapyrrole pathway, and its level is regulated at the transcriptional and post-transcriptional level (Tanaka and Tanaka, 2007; Tanaka et al., 2011). The biosynthetic pathways of chlorophyll and heme follow the same pathway up to protoporphyrin IX (proto IX) where they split in two branches, the chl branch and the heme branch (Mochizuki et al., 2008).

In green algae and higher plants disruption of the tetrapyrrole pathway impacts the expression of photosynthesis-associated nuclear genes (*PhANGs*) (Strand et al., 2003; von Gromoff et al., 2008; Zhang et al., 2011). Furthermore, several studies were conducted with *Arabidopsis gun* mutants (*genomes uncoupled*) that partially recovered *LHCB1* expression following norflurazon (NF) treatment (Susek et al., 1993). When exposed to oxidative stress, the *gun1-6* mutant expressed *PhANGs*, whereas these genes were repressed in the WT supporting the evidence that some tetrapyrrole intermediates are involved in the communication network between chloroplasts and nucleus. Heme and Mg-ProtoIX affect *PhANGs*, in particular those of proteins involved in the primary reactions of photosynthesis, Calvin cycle enzymes, and enzymes of the tetrapyrrole biosynthetic pathway.

At the branchpoint Mg^2+^ or Fe^2+^ are inserted into Proto IX by Mg-chelatase (MgCh) or ferrochelatase (FC) to produce Mg-protoporphyrin IX (MgProto) and heme, respectively (Figure 5). In plants, MgCh consists of three subunits: CHLI, CHLD, and CHLH. CHLD and CHLH are encoded by a single gene in *Arabidopsis*, while CHLI exists in two isoforms, CHLI1 and CHLI2. CHLI1 is required for photosynthesis (Koncz et al., 1990; Rissler et al., 2002), whereas CHLI2 only plays a minor role in the assembly of MgCh (Kobayashi et al., 2008). Furthermore, GUN4 increases MgCh activity by regulating substrate or product channeling (Larkin et al., 2003; Shimizu and Masuda, 2021).

Analysis of *gun* mutants of *Arabidopsis* and mutants affected at different steps of the tetrapyrrole pathway in Chlamydomonas suggests that tetrapyrroles have a regulatory role in plastid signaling in plants and algae and are involved in retrograde signaling. The chlorophyll biosynthesis inhibitor a, *a*-dipyridyl was reported to limit light-induced *LHCB* mRNA increase in Chlamydomonas. This compound blocks the chlorophyll biosynthetic pathway at a late step which leads to the buildup of Mg-protoIX methyl ester (Mg-ProtoIX-ME) (Johanningmeier and Howell, 1984; Kleine et al., 2009). Mg-protoIX methyl ester is a porphyrin intermediate that is used as a marker for environmental changes that affect *PhANG* expression (Kindgren et al., 2011). When the chlorophyll biosynthetic pathway was disrupted upstream of Mg-ProtoIX, the above-mentioned inhibition of chlorophyll biosynthesis did not occur. This suggests that Mg-ProtoIX and Mg-ProtoIX-ME may act as negative factors in chlorophyll biosynthesis and are needed for the repression of *LHCB* transcription (Jasper et al., 1991). In addition, Mg-ProtoIX and Mg-ProtoIX-ME are required for the induction of HEAT SHOCK PROTEIN 70A/B (HSP70A and HSP70B), the cytosolic and plastid-localized proteins, respectively, in Chlamydomonas (Johanningmeier and Howell, 1984; Fortunato et al., 2022; Li et al., 2022). According to Vasileuskaya et al. (2004), Mg-ProtoIX also stimulates *HEMA*, which encodes GluTR in Chlamydomonas (Figure 5). Light induction of HSP70 is prevented in the chlorophyll-deficient *brs-1* mutant affected at a step upstream of Mg-ProtoIX (Vasileuskaya et al., 2004; Toyokuni et al., 2022). Also Mg-ProtoIX buildup is needed for HSP70 light induction in the Chlamydomonas mutant *PC-1/Y-7* which is unable to convert protochlorophyllide to chlorophyllide in the dark (Hess et al., 1994).

Recently, a T-DNA insertion was found 8 kb upstream of the gene *ferrochelatase I* (*FC1*) encoding chloroplast heme synthase/plastid ferrochelatase in a *gun* mutant called *gun6-1D*. It has the same phenotype as the *gun2-gun5* mutants with higher expression of *PhANGs* than in WT under photooxidative conditions (Woodson et al., 2013). In this mutant *FC1* is over-expressed. It was proposed that heme accumulates in *gun6-1D* and appears to be a positive retrograde signal, as evidenced by increased flux through the heme branch of the tetrapyrrole biosynthesis pathway and increased expression of *PhANGs* in the mutant (Voss et al., 2011; Woodson et al., 2013; Figure 5).

Ferrochelatase inserts Fe^2+^ into Proto IX in the heme branch to form protoheme (heme b), the prosthetic group of *b*-type cytochromes and of proteins like catalase and peroxidase. In *Arabidopsis* and cucumber, there are two isoforms of FC (*FC1* and *FC2*) which have different tissue-specific and developmental expression profiles: *FC2* is light-dependent and mostly expressed in photosynthetic tissues, whereas *FC1* is stress-responsive and found throughout the plant (Chow et al., 1998; Nagai et al., 2007). Other hemes, such as heme *a* and heme *c*, are synthesized from protoheme. Bilins use protoheme as a substrate. Heme oxygenase oxidizes protoheme to produce biliverdin IX. Phytochromobilin synthase then transforms biliverdin IX into 3Z-phytochromobilin. Finally, 3Z-phytochromobilin is isomerized to 3E-phytochromobilin, which serves as the chromophore of phytochromes (PHY) (Terry et al., 2002; Shimizu and Masuda, 2021).

Recent studies with Chlamydomonas have revealed novel essential functions of bilins in retrograde signaling, notably in the regulation of the expression of genes involved in the detoxification of ROS (Duanmu et al., 2013, 2017). Moreover bilins also function in the maintenance of a functional photosynthetic apparatus as well as in the regulation of chlorophyll synthesis by binding GUN4, an important regulator of Mg chelatase (Zhang et al., 2021).

#### Plastid gene expression pathway and retrograde signaling

Retrograde signaling is triggered when PGE is disrupted at the transcriptional or translational levels (Woodson and Chory, 2008). PGE is responsible for changing *PhANG* expression (Zhang et al., 2011). PGE is also closely regulated by redox signals from the thylakoid membrane, which are transmitted through an intricate network of phosphorylation processes (Pfannschmidt et al., 2001; Pfannschmidt and Liere, 2005; Pfannschmidt, 2010). Use of the PGE inhibitors chloramphenicol and lincomycin (LIN) revealed the role of PGE in retrograde signaling (Stern et al., 2010; Pogson et al., 2015; Figure 6). When chloramphenicol or LIN is applied to immature seedlings, *PhANGs* are repressed. The retrograde signal from PGE was found to be light-independent in two photomorphogenic mutants, *cop1-4* in *Arabidopsis* and *lip1* in pea which partially restore *LHCB1.2* expression in the dark compared to wild type (WT) upon LIN treatment (Sullivan and Gray, 1999; Tanaka and Tanaka, 2007). Sigma factors have also been shown to influence PGE *in vivo* through reversible phosphorylation. In addition, the redox state of chloroplasts controls *SIG1* activity, which impacts the expression of the *psaA* gene, implying a link between photosynthetic activity and *SIG1* phosphorylation, as well as the expression of photosynthesis genes possibly via the *Sensor Kinase* (*CSK*) in chloroplasts (Shimizu et al., 2010; Liebers et al., 2020). A nucleus-encoded circadian oscillator regulates PGE cycles. *SIG5* which regulates the transcription of a number of chloroplast genes in *Arabidopsis* (Shimizu et al., 2010; Noordally Zeenat et al., 2013) is also involved in this circadian gating of light input to chloroplast genes (Noordally Zeenat et al., 2013).

Several components involved in the signaling pathway have been identified (Figure 6). GUN1, a plastid-localized pentatricopeptide repeat protein, is one of the key regulators in plastid-to-nucleus signaling (Koussevitzky et al., 2007). Signals from at least four distinct plastid processes (tetrapyrrole biosynthesis, PGE, redox, and plastid protein import) have been found to be mediated by GUN1 (Kozaki et al., 2000; Koussevitzky et al., 2007), although the links between GUN1 and other components within plastids such as STN7 remain unclear. Two nuclear transcription factors, ABI4 and GLK1, may regulate the expression of photosynthetic genes downstream of GUN1 (Figure 6). ABI4 is a negatively acting transcription factor in response to plastid signals. According to the current model, ABI4 competitively binds to the G-box *cis*-element in response to plastid signals and inhibits the induction of *LHCB* expression (Koussevitzky et al., 2007). GUN1 activates ABI4 through an as yet uncharacterized mechanism which is also used by signals arising from tetrapyrrole biosynthesis, PGE, and redox status (Figure 6). However, the signal arising from protein import defects does not use this pathway. Instead, it involves GLK1 (Kozaki et al., 2000). Unlike ABI4, GLK1 is a positive regulator of *LHCB* expression. GLK1 coordinates the expression of photosynthesis related genes in the nucleus (Nakamura et al., 2009; Waters et al., 2009). When plastids are damaged by stresses such as NF treatment or a protein import defect, the expression of *GLK1* is significantly repressed, leading to subsequent down-regulation of photosynthetic genes. Furthermore, GUN1 activity is necessary for the repression of GLK1 (Kozaki et al., 2000). The level of GLK1 also appears to be regulated at the protein level (Waters et al., 2008). Hence unraveling the mechanism by which GLK1 expression/accumulation is regulated may be one of the key tasks for the near future.

In mustard, the retrograde signaling system is active exclusively at an early developmental stage of seedlings (Nott et al., 2006). Analysis of the *Arabidopsis prors1* mutant, on the other hand, suggests that the PGE pathway is maintained in mature leaf tissue (Pesaresi et al., 2006, 2009). The *Arabidopsis prors1* mutant is deficient in prolyl-tRNA synthetase leading to reduced protein synthesis in both the plastids and mitochondria. *PhANGs* are specifically down-regulated in mature *prors1* plants (Pesaresi et al., 2006). This decreased expression, however, was not observed in the *Arabidopsis mrpl11* or *prpl11* mutants, which are deficient in the mitochondrial and plastid ribosomal L11 proteins, respectively (Pesaresi et al., 2007, 2011), but was observed in the *mrpl11 prpl11* double mutant. This finding indicates that plastid and mitochondrial translation act cooperatively to control *PhANG* expression (Pesaresi et al., 2011). The analysis of transcriptionally active plastid chromosomes (TAC) has recently shown that additional proteins are necessary for chloroplast transcription besides the main components of PEP (Pfalz et al., 2006). TRXz and its intrinsic partners fructokinase-like protein 1 (FLN1) and 2 (FLN2) are associated with the plastid-encoded RNA polymerase (PEP), suggesting that these proteins mediate the redox regulation of PGE (Steiner et al., 2009, 2011; Stern et al., 2010; Wimmelbacher and Börnke, 2014). Studies of the *trx-z Arabidopsis* mutant revealed alterations in PGE that are diagnostic for PEP deficiency, suggesting that TRX-z is required for PEP function. Furthermore, Wimmelbacher and Börnke (2014), demonstrated that TRX-z and FLN1 are structurally important components of the PEP complex, and that the fine-tuning of PEP function may be controlled by TRX-z and FLN1 redox activity (Chan et al., 2014; Wimmelbacher and Börnke, 2014).

#### Role of singlet oxygen in signaling from plastids to nucleus

Chloroplasts are very important sensors of environmental factors such as high light, low or high temperature, and drought that perturb photosynthetic electron transport (Baruah et al., 2009a,b). Under these stress conditions, light energy absorbed by the photosynthetic apparatus may exceed its capacity and lead to an over-reduction of the electron transport chain (Kramarenko et al., 2006). Photosynthetic organisms have developed several ways to dissipate excess light excitation energy to avoid this over-reduction which can produce damaging ROS (Blazek et al., 1989; Baruah et al., 2009a). Oxygen is chemically inactive in its ground state, but it can be turned into ROS through light-induced electron transfer reactions. Different forms of ROS can be formed such as superoxide, hydrogen peroxide (H_2_O_2_), hydroxyl radicals and singlet oxygen (^1^O_2_) (Carloni et al., 1993). Depending on the amounts of these ROS produced, they can cause oxidative damage or act as signaling molecules.

Conversion of protochlorophyllide to chlorophyllide is a key step in chlorophyll synthesis and requires light in land plants. Protochlorophyllide is highly photodynamic and its accumulation in the dark causes photo-oxidative damage when plants are shifted from the dark to the light. This is prevented by the FLUORESCENT (FLU) protein which inhibits glu-tRNA reductase, an enzyme which catalyzes one of the early steps of chlorophyll synthesis (Kauss et al., 2012; Figure 5). After a shift form darkness to light, mutants deficient in FLU produce ^1^O_2_ and initiate a programmed cell death (PCD) response and photobleach (Meskauskiene et al., 2001). However, these mutants survive in constant dark or constant light. A suppressor screen of the *flu* mutant in *Arabidopsis* led to the identification of the chloroplast proteins Executer 1 (EX1) and its homolog EX2. In the *ex1* suppressor the ^1^O_2_-induced programmed cell death response no longer occurs (Wagner et al., 2004; Lee et al., 2007; Pruzinská et al., 2007). EX1 and EX2 play a critical role in this cell response to a burst in ^1^O_2_ which elicits changes in gene expression (Figure 6; Brzezowski et al., 2012; Prabha et al., 2016). Further screening was performed with a transgenic *flu* line containing a reporter driven by the promoter of an ^1^O_2_-responsive AAA-ATPase gene for constitutive high-level expression. One of the mutant isolated is affected in the *Pleiotropic Response Locus 1* (*PRL1*) gene. This gene appears to suppress the expression of ^1^O_2_-responsive genes and to play an important role in the response of plants to environmental stress. Additional genetic studies also suggest that ^1^O_2_-mediated signals are transmitted to the nucleus through a signaling network and that ^1^O_2_-mediated retrograde signaling interconnects to other signaling pathways (Baruah et al., 2009a,b).

Other ^1^O_2_-response mutants (*caa33, soldat8, and soldat10*) are similarly deficient in mechanisms that impact plastid homeostasis, resulting in an increased ^1^O_2_ response (Lee et al., 2007; Simková et al., 2012). All of these examples show that the response to ^1^O_2_ in plants is part of a complex signaling network that is influenced by other signaling pathways (Baruah et al., 2009b). ^1^O_2_ signaling affects gene expression not only at the transcriptional level, but also at the translational level (Simková et al., 2012). In response to ^1^O_2_, the barley *flu*-orthologous mutant *tigrine-d.12* had decreased expression of a number of chloroplast proteins (Khandal et al., 2009). This inhibition of protein synthesis was due to a halt in translation initiation on 80S cytoplasmic ribosomes which correlated with a decrease in phosphorylation of the ribosomal protein S6 (Liebers et al., 2020).

The *HSP70A* gene was shown to be induced by ^1^O_2_ and several *HSP70A*-based reporter constructs reacted specifically to either ^1^O_2_ or H_2_O_2_ in *Chlamydomonas.* The *HSP70A* promoter region is composed of two promoters named *P*_*A*1_ and *P*_*A*2_, two separate cis-acting regulatory regions, that confer inducibility by hydrogen peroxide and singlet oxygen (Shao et al., 2007). The *GPXH/GPX5* gene is significantly induced by ^1^O_2_, but only weakly increased by other ROS, and it is selectively induced by ^1^O_2_ under high light (Fischer et al., 2009, 2010). In addition, the transcriptional activation of *GPXH* is dependent on several regulatory factors different from reactive electrophile species (RES). RES signaling, another ^1^O_2–_specific response, is likely to be involved in Rose Bengal (RB) acclimation in *C. reinhardtii*. This ^1^O_2_ specific response includes the strong induction of *GPXH* and maybe other genes at low RB concentrations and during the early high light response (Fischer et al., 2010). A reporter construct containing a portion of the ^1^O_2_-specific GPXH promoter was utilized to test over 5,000 UV-mutagenized clones for their responsiveness to ^1^O_2_. The analysis of these mutants revealed that, similar to plants, the ^1^O_2_ signal is not conveyed along a single linear chain in *C. reinhardtii*, as no mutation defining signal transmission could be isolated (Fischer et al., 2006). *PSBP2* is a paralog of the oxygen-evolving enhancer gene *PSBP1*, and it has been proposed that it functions as an ^1^O_2_ responsive factor to activate *GPXH* expression. However activation of the wild-type *GPXH* gene and adaptation to ^1^O_2_ are not completely abolished in *psbP2*. This suggests that different signaling pathways are involved in the response to ^1^O_2_ in *C. reinhardtii* (Figure 6; Fischer et al., 2010; Brzezowski et al., 2012).

### Additional possible plastid signaling molecules

Although several retrograde signaling chains have been identified, the nature of the plastid retrograde signaling molecules is still largely unknown. Norflurazon (NF), an inhibitor of carotenoid synthesis has been used extensively for studying retrograde signaling. Upon treatment of seedlings with NF and exposure to light, they bleach because photooxidation of chlorophyll in the absence of carotenoids leads to the accumulation of tetrapyrrole intermediates that are highly photodynamic. Under these conditions expression of *LHCB* genes is strongly decreased in wild-type plants. Mg-proto IX levels are 15 times higher in NF treated compared to untreated *Arabidopsis* seedlings (Strand et al., 2003; Chan et al., 2014; Garmash, 2022). When seedlings were fed tetrapyrrole intermediates, such as heme, porphobilinogen, or proto IX, they did repress *LHCB* promoter-driven expression of luciferase (Strand et al., 2003). Expression of *LHCB* was repressed by feeding Mg-proto IX directly to leave protoplasts implying a specific control of expression of *PhANGs* by Mg-proto IX (Strand et al., 2003). Mg-proto IX increased in both plastids and cytosol under stress (Strand et al., 2003; Tyagi and Gaur, 2003). However these claims have been questioned by other studies which showed no correlation between tetrapyrrole intermediates, including Mg ProtoIX, and *LHCB* expression (Mochizuki et al., 2008; Moulin et al., 2008). It is thus unlikely that Mg-proto IX is exported from the plastid to the cytosol as a retrograde signal. The signal created by tetrapyrrole intermediates might also be produced from ROS (Ankele et al., 2007), either directly or indirectly, or derived from redox signals, because perturbation of the tetrapyrrole biosynthetic pathway could result in ROS formation or changes in the plastid redox state (Surpin et al., 2002; Mochizuki et al., 2008, 2010; Monaghan et al., 2009).

Another tetrapyrrole intermediate, heme, affects nuclear gene expression and appears to be involved in retrograde control. In the *Arabidopsis gun6 gain-of-function* mutant, heme was found to be a retrograde signaling molecule (Estavillo et al., 2011). MEcPP (methylerythritolcyclodiphosphate) and PAP (3’-phosphoadenosine 5’-phosphate) two key metabolites generated in plastids that have been proposed to act as signaling molecules in the nucleus, in addition to the tetrapyrrole intermediates. PAP accumulates in the plastid under stress conditions, although it has no known physiological role in plant cells (Estavillo et al., 2011; Xiao et al., 2012; Wu et al., 2019). Although PAP is generated in the plastids, expression of the phosphatase SAL1 in the nucleus lowers overall PAP levels (Estavillo et al., 2011). PAP appears to regulate gene expression by altering RNA metabolism mediated by 5’-3’ exonucleases. MEcPP, like PAP, plays a vital function in retrograde signaling, although it is unknown how it moves from the plastid to the nucleus. The concept of a metabolite signal makes sense since the plastid constantly conveys its metabolic state to the cytosol through the interchange of various metabolites, which ultimately leads to changes in nuclear gene expression (Pfannschmidt, 2010; Estavillo et al., 2011; Leister and Kleine, 2011; Xiao et al., 2012). As a result, a number of metabolites, including carbohydrates and redox valves, have been proposed to be retrograde signals (Kleine et al., 2009; Leister, 2012). It should be noticed that these new signals were discovered during physiologically relevant stress conditions like drought or high light unlike the previously identified signals induced through artificial stress conditions.

Reactive oxygen species molecules have also been proposed to function as retrograde signaling molecules as they have the ability to move across the subcellular compartments, which is one of the main characteristics of the signaling molecules. H_2_O_2_ may be the best candidate whereas ^1^O_2_ is thought to have a less important role because of its short half-life, high reactivity and limited diffusion (Nott et al., 2006; Larkin and Ruckle, 2008; Leister, 2012; Maruta et al., 2012; Li and Kim, 2022). β-Cyclocitral (β-CC), a ^1^O_2_ oxidation product of carotenoids was identified in *Arabidopsis* as a messenger in the ^1^O_2_ signaling pathway and induces gene expression in response to ^1^O_2_ (Ramel et al., 2012; Figures 5, 6). As it is volatile, β-CC has the ability to travel through the cytosol by diffusion (Leister and Kleine, 2011; Leister, 2012; Liang et al., 2022), to regulate nuclear gene expression and to act as a downstream messenger for rising levels of ^1^O_2_ (Ramel et al., 2012).

## Conclusion and future perspectives

Plastids are essential compartments of eukaryotic cells. They are involved in the synthesis of many metabolites and are the sites of important metabolic and regulatory pathways. Here we have discussed several retrograde-signaling pathways that are highly complex and diverse. They are not operating in every cell type but restricted to specific cell types and developmental stages. As an example, the singlet-oxygen–mediated pathway occurs mostly in differentiated photosynthetically active mesophyll cells. In the last few decades, significant advances have been achieved in our understanding of plastid-to-nucleus signaling. However, in spite of this progress, there are still a lot of question marks concerning the identity of the authentic retrograde signals and how they work. Possibly, signals could only accumulate transiently and locally and be therefore difficult to detect. It is also not clear how perturbations of chloroplast processes elicit retrograde signals and how these signals are transmitted from the plastids to the nucleus and how they change the expression of nuclear genes. For a long time, the redox state of the plastids and PGE were thought to be the main determinants of retrograde signaling. Yet we still do not know how changes in redox state or impairment of PGE are transduced into signals and their identity remains elusive. At this time, it is not clear whether the signals are proteins, proteolytic products, oxidized lipids, metabolites or ionic fluxes.

Most of the cellular biosynthetic pathways occur, at least partly, in the plastids, and specific carriers move different metabolites into and out of them. Different retrograde signaling pathways have been proposed based on different approaches and different experimental models. But it is not yet clear how these signaling networks interact with each other and how some key transcriptional factors like PEND, GLK, WHIRLY1, AP2/EREBP operate within these networks. Clearly, these challenging questions will need to be addressed in the coming years.

## Author contributions

XS designed the review outline and wrote the “Conclusion and future perspectives” section of the manuscript. MJ, ZL, and J-DR wrote the other sections and designed all figures. All authors contributed to the article and approved the submitted version.
